# An Elderly Care System Based on Multiple Information Fusion

**DOI:** 10.1155/2018/4098237

**Published:** 2018-01-15

**Authors:** Zhiwei He, Dongwei Lu, Yuxiang Yang, Mingyu Gao

**Affiliations:** College of Electronic Information, Hangzhou Dianzi University, Hangzhou, China

## Abstract

With the development of social economy in the 21st century, and the rising of medical level, the aging of population have become a global trend. However lots of elderly people are in “empty nest” state. In order to solve the problem of high risk of daily life in this group, this paper proposed a method to integrate the information of video images, sound, infrared, pulse, and other information into the elderly care system. The whole system consists of four major components, that is, the main control board, the information acquisition boards, the server, and the client. The control board receives, processes and analyzes the data collected by the information acquisition boards, and uploads necessary information to the server, which are to be saved to the database. When something unexpected occurs to the elderly, the system will notify the relatives through the GPRS (general packet radio service) module. The system also provides an interface for the relatives to inquire the living status of the elderly through an app. The system can monitor the living status for the elderly with the characteristics of quick response, high accuracy, and low cost and can be widely applied to the elderly care at home.

## 1. Introduction

The aging of population is a global issue [1], especially in China. Most children who are busy with their work have little time to take care of their parents and have a great pressure on parent support. As most elderly become empty nesters, monitoring the living status of them is to solve not only family problems but also social problems. China's welfare science for elderly is still in the early stages of development [2, 3]. The existing products based on wearable sensors sometimes feel inconvenient and are easy to forget to be carried. The products based on audio sensor can judge the living condition of the elderly through sound signals, but they are vulnerable to environmental noise, which leads to low accuracy. The products based on vision sensors also have some problems such as limited visual acquisition and privacy leakage. Therefore, developing an elderly care and monitoring system which meets the privacy protection requirement has great significance in family, social, and practice value. The system should be able to effectively monitor the daily life and correctly assess the health status of the elderly. When something unexpected happens, the system will send an alarm signal to inform the family relatives or other related people.

Some related systems have been proposed in the literature [4–8]. For example, Kidd et al. [6] proposed the “Aware Home” system, which captured real-time images of the elderly through the camera, and the children can see the elderly current activity information through the Internet and can view the recorded information to better understand the status of the elderly. Recently, Khosla et al. [7] reported an interactive multimodal social robot system for improving quality of care of elderly in Australian nursing homes. In their system, they utilized multimodal interaction (voice, gestures, emotion, touch panel, and dance) in assistive social robot. Suryadevara and Mukhopadhyay [8] proposed a wireless sensor network-based home monitoring system for wellness determination of elderly. In their system, they used a number of sensors interconnected to detect usage of electrical devices, bed usage and chairs along with a panic button, and wireless sensor network consisting of different types of sensors like electrical and force, and contact sensors with Zigbee module sensing units are installed at elderly home.

In this paper, we propose an elderly care system based on multi-information fusion technology, using video processing technology as the core, combined with sound detection, infrared detection, and pulse detection. Specifically, the proposed system uses a DSP + ARM dual-core board, with OMAP L138 as the processor. A six-layer PCB is applied and designed in order to simplify the circuit and reduce the cost. Through the method of background modeling and updating, the foreground moving human being can be extracted properly with the help of an 8-connectivity analysis and shadow removal. Using some features of the minimum circumscribed rectangle, the falling-down of the elderly can be detected properly. In order to obtain the living information of the elderly without privacy disclosure, several information acquisition boards armed with infrared sensors, laser sensors, sound sensors, and pulse sensors are used at the gate, in the toilet, and in the bedroom. The multiple information fusion mechanism improves the efficiency and accuracy of the elderly care system. Hence, the developed system can accurately track the indoor position of the elderly, detect abnormal activities, and inform the relatives automatically when something unexpected happens. In general, this research undoubtedly provides important basis for the generalization and application of the elderly care system.

The rest of the paper is organized as follows. In Section 2, the whole system is described in detail. In Section 3, the video analysis-based falling-down detection algorithm is given. System setup and experimental results go to Section 4. Finally, in Section 5, the conclusions and discussions are given.

## 2. System Description

### 2.1. System Overview

**Figure 1:**
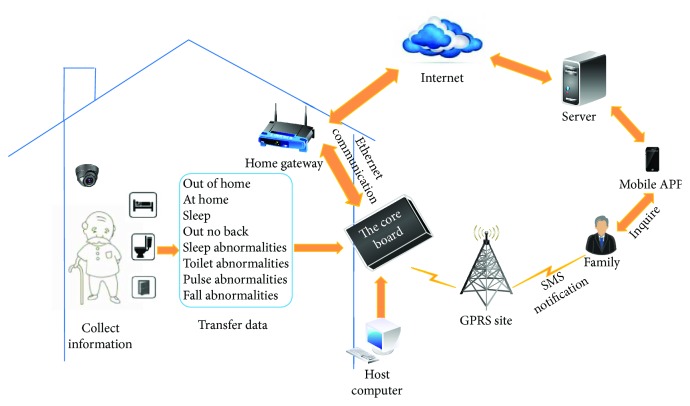
Schematic diagram of system.

The schematic diagram of the whole system is shown in Figure 1. The whole system consists of a main board and several information acquisition boards. The main control board is the core of the hardware system, while the information acquisition boards are the basis. The information acquisition boards are installed around the room at the right places. The voice, infrared, and pulse data are then collected directly by these information acquisition boards and some of the living status of the elderly, such as whether he/she is absent or is sleep abnormally, can then be obtained easily. The living status whether the elderly falls down is obtained through video analysis by the main board. When all these living conditions are obtained, they are then uploaded to the server through Ethernet. Relatives can view the real-time status and historical status of the elderly with his/her mobile phone through a special installed app on it. On the other hand, when something unexpected occurs and is detected by the system, a short message will be sent automatically to the relatives through a GPRS (general packet radio service) module installed on the system.

### 2.2. Design of the Hardware System

**Figure 2:**
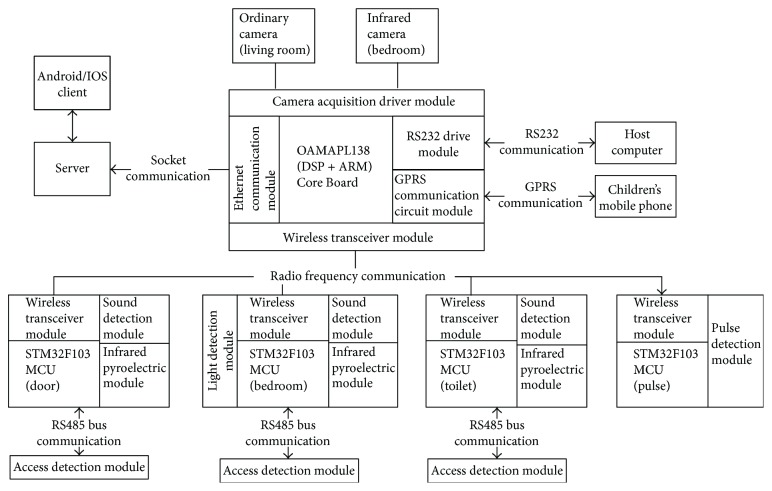
Schematic diagram of the hardware platform.

For the hardware design, three aspects should be considered. First, the hardware system works in an indoor environment, and the main influence is the temperature and weather change. Second, the cameras are fixedly installed, so the video analysis algorithm has a certain robustness to simple noise interference. Third, the system should work in real-time. According to these aspects, we build the hardware platform based on a DSP (digital signal processor) + ARM (acorn RISC machine) dual-core CPU (central processing unit) with OMAP (open multimedia application platform) L138 [9] as the processor which is developed by Texas Instruments, along with three infrared and sound detection modules (information acquisition control panel), one pulse detection module, and two analog cameras as the sensors. The hardware platform has powerful data processing capabilities to meet the system's real-time and efficiency requirements.  Figure 2 shows the diagram of the system hardware platform.

1. The core of the main board, OMAP L138, is a dual-core (DSP + ARM) CPU with an up to 456 MHz working frequency, a 512 MByte extended NAND Flash and a 128 MByte DDR2 (double data rate 2) memory, and a wealth of external interfaces such as the Ethernet, video, and LCD (liquid crystal display) interface, and so forth. The main control board utilizes the wireless transceiver module YB30_SI4432 which is developed by Silicon Labs (http://www.silabs.com) to communicate with the information acquisition board, which has the characteristics of long transmission distance, low cost, high integration, and high ability going through a wall. The living conditions and the captured images when the elderly falls down are sent through this module.
2. There are four pieces of information acquisition board which uses the microcontroller STM32F103 [10], which is developed by ST Microelectronics, as the core. Three of them, armed with infrared, sound, and laser detection modules, are installed in the toilet, the bedroom, and the gate, respectively, to detect abnormal conditions including long-time staying in the toilet, not returning back home, or sleeping disorders. As mentioned before, once abnormality occurs, data will be transmitted to the main control board through the wireless transceiver module. The last one piece is used to detect the elder's pulse frequency. It can be carried with the elderly all the time or measured when needed.
3. The main control board also judges abnormal status in the living room through an analog camera when the elderly falls down or detects abnormal sleeping status in the bedroom through another infrared camera.
4. The dual-core main control board will send the collected information to the server through the Ethernet transmission. The server will save the data to a database and show the current status of the elderly.

### 2.3. Design of the Software System

**Figure 3:**
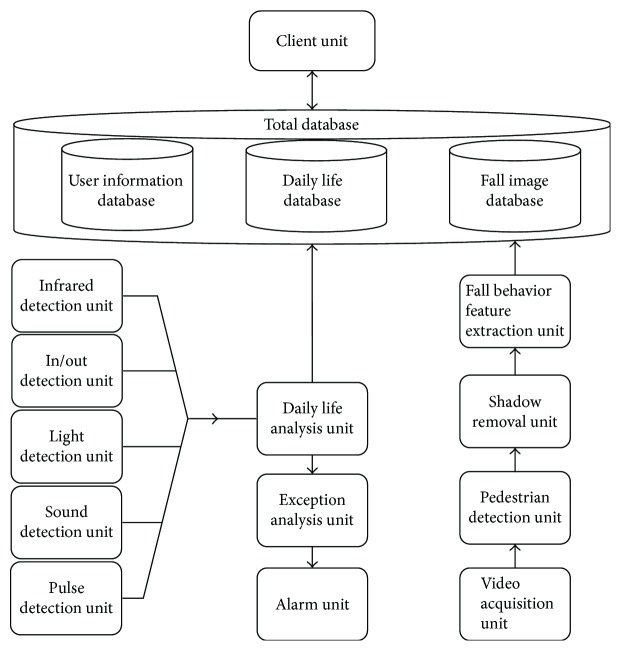
Schematic diagram of the system software.

In this paper, the software system consists of four parts: the main control board software, the information acquisition board software, the server software, and the client software. The functions of the main control board software include abnormal status judgment, notice warning, and fall detection. The functions of the information acquisition board software include infrared detection, laser detection, sound detection, and pulse detection. The main function of the server software is to do database operations, and the client software is the interface for relatives to view/review the status of the elderly. The diagram for the whole software is shown in Figure 3.

#### 2.3.1. Design of the Main Control Board Software

**Figure 4:**
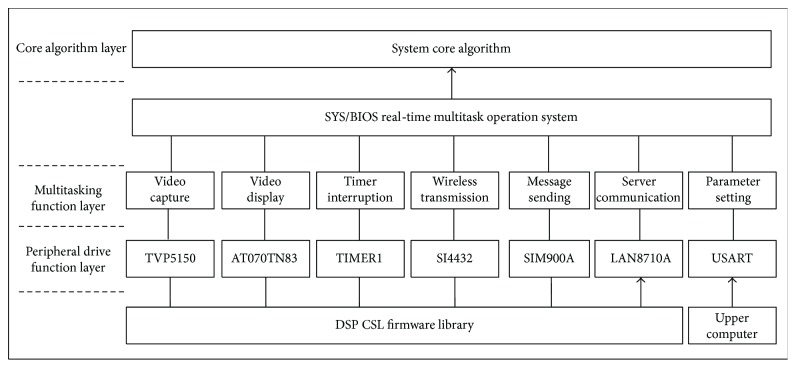
Software framework of the main control board.

The software of the main control board is divided into three layers from the bottom to the top: the peripheral driver function layer, the multitask function layer, and the core algorithm layer. The driver function layer is used to initialize the peripheral of the main control board with CSL (chip support library) firmware library to drive them to work normally. The multitask function layer is designed to realize different tasks of the system, including the video capture, video display, timer interrupt, wireless transceiver, SMS sending, and server communication tasks. The core algorithm layer mainly accomplishes the fall detection of the elderly. The software framework of the main control board is shown in Figure 4.

**Figure 5:**
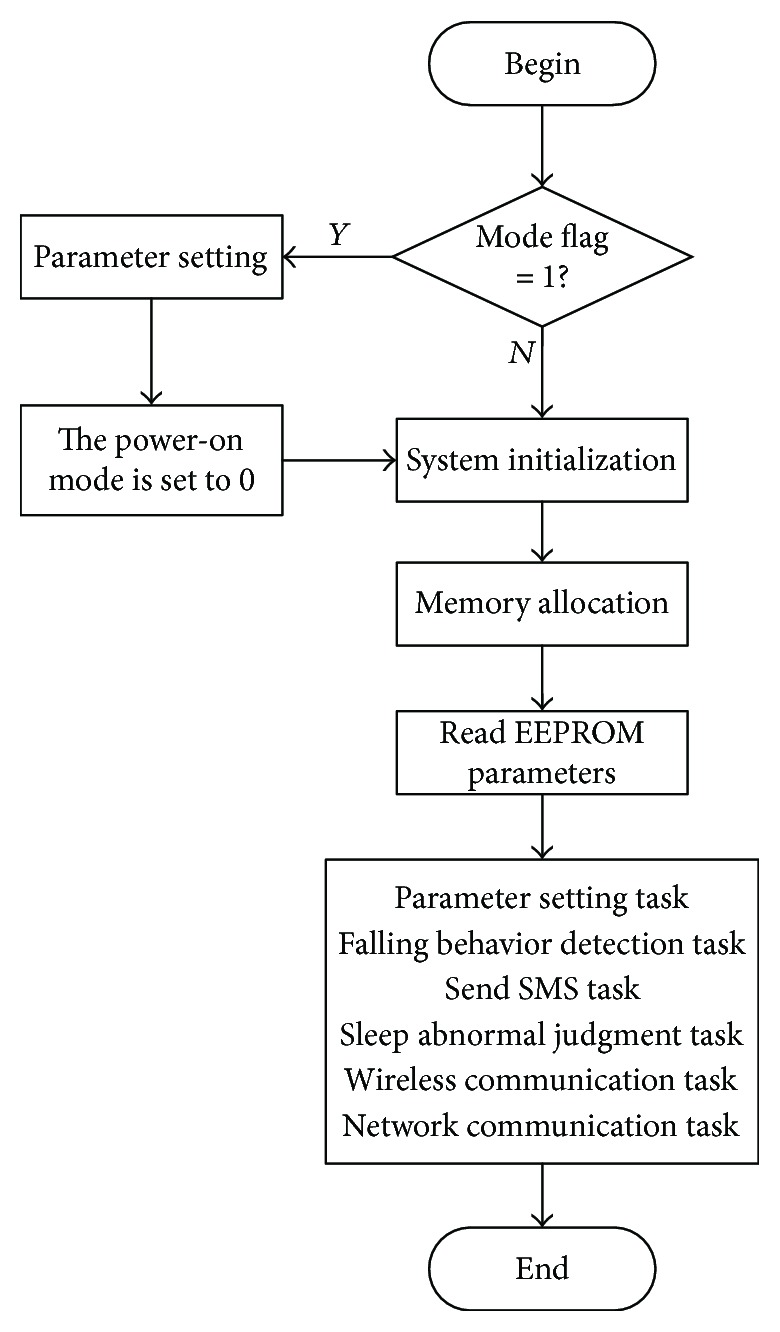
Software process of the main control board.

According to the software framework and combined with the characteristics of the SYS/BIOS multitasking OS (operating system), the software workflow of the main control board is shown in Figure 5.

After the main board is powered on, the system will first do some initialization work such as system clock configuration, CSL library initialization, and peripheral and memory resource initialization. The details are as follows:

1. System clock configuration: setting OMAP L138 system clock to 456 MHz
2. Peripheral initialization: GPIO (general purpose input/output) port initialization, LAN8710A's driver initialization, EEPROM initialization, SI4432's driver initialization, TIMER1's driver initialization, TVP5150's driver initialization, AT070TN83's driver initialization, timer SIM900A's driver initialization, and RS232's driver initialization
3. Memory resource management: mapping the appropriate data to DDR2
4. Read and set the parameters in EEPROM
5. Multitask operation: parameter setting tasks, falling behavior detection tasks (including target detection, target tracking, and falling behavior recognition), SMS task, sleep abnormality judgment task, wireless communication, and network communication task

The “Mode Flag” in Figure 5 is set through the hardware DIP switch. When the mode flag “ModeSet” is 1, the system is set at parameter setting mode, in which mode the manager can modify a series of parameters such as the room number, the mobile phone number and the threshold in the video analysis algorithm, or even the IP address through PC software. When the “ModeSet” is 0, the system is set at working mode.

#### 2.3.2. Design of the Information Acquisition Board Software

As mentioned before, we use the information acquisition board to collect and judge abnormal living information at the gates, toilets, and bedrooms.

**Figure 6:**
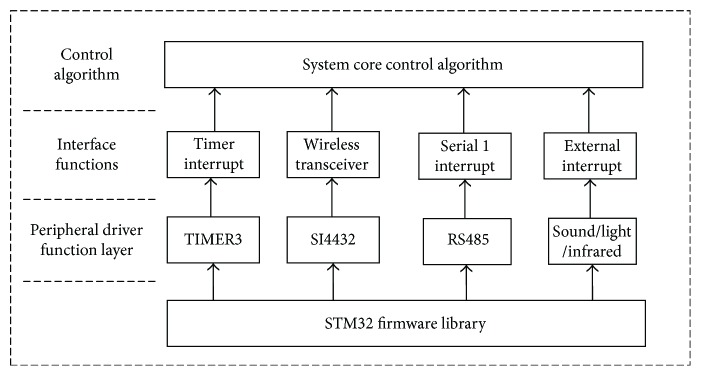
Software framework of the Information acquisition board.

The software diagram of the information acquisition board is shown in Figure 6. It has three layers, that is, the peripheral driver function layer, the interface function layer, and the control algorithm layer. The peripheral driver function layer initializes and drives peripherals of the STM32. The timer TIMER2 is initialized as a general timer, which generates an interruption every 1 second to receive and determine the various signal and changes from sensors. The IN/OUT detection module communicates with the STM32 through the RS485 bus and received the IN/OUT status of the elderly through a serial port interrupt. The sound, light, and infrared sensors receive data through an external interruption and jointly judge the elderly living status. The SI4432 wireless module connects with the STM32 through the SPI bus interface for data communication. The board will send the elderly status information to the main control board.

The three living information of the elderly at the gate that should be detected include out (01), at home (02), and out without going home (03); the three living information of the elderly in the bedroom include getting up (04), sleeping (05), and sleep abnormality (06); the toilet abnormality (07) is the only a status that should be detected in the toilet. Each status is obtained through multiple sensors fusion.

Let us take the information collected at the bedroom as an example to explain the logic of the information fusion system:

1. An entrance detection sensor detects that the elderly enters the bedroom.
2. If the light sensor module detects that the elderly on the bed is moving, and the current time is the sleeping time of the elderly, the system judges that the elderly starts to sleep.
3. When the elderly is sleeping, but the sound and infrared sensors cannot detect any effective data for more than 20 seconds, the system would suspect that sleep abnormality occurs.
4. Within the sleeping mode, the entrance detection sensor module detects effective data, and if the current time is the wake-up time, the system would judge that the elderly gets up.

#### 2.3.3. Design of the Client Software

The client software can be divided to two parts, the PC (personal computer) monitoring client and the mobile phone client.

**Figure 7:**
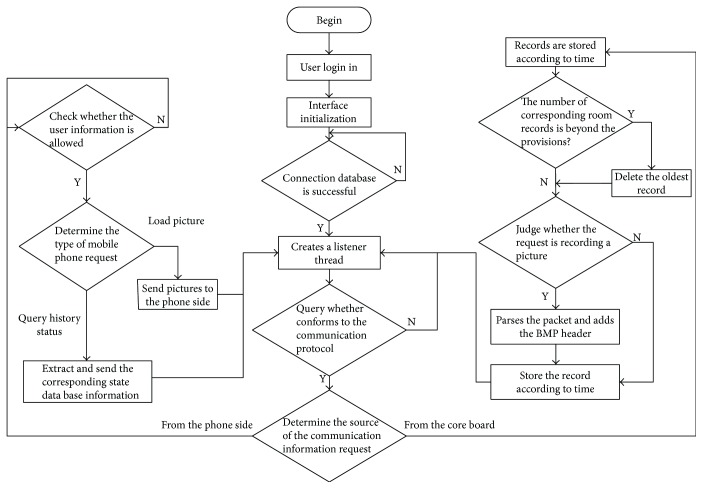
Client software flow chart.

1. The PC monitoring client displays the data from the database server, including the living status of the elderly received from the main board and the abnormality along with the time of occurrence. The PC end software is developed in Visual Studio 2013 with the development language of C# and the database of SQLSever2012.
2. In order to adapt to the most widely used two mobile phone operating systems, that is, the Android [11] and the iOS, two mobile phone clients are developed, respectively. The Android client software development platform is eclipse4.5, with the development language JAVA, and the iPhone client development platform is Mac OS X, with the language Objective-C. The mobile phone client includes a login interface, a status interface, and a message list interface. Users need to enter the correct username and password to login, which protect the privacy of the elderly to some extent. The server will record all the information into the database; when users log on to the mobile client to query the current status or images of the elderly, the server will package them in a standard Json format and send them to the mobile phone, and the mobile client will then parse the received package and print the message list for the users to view. If a falling-down event occurs, the relatives can view the falling-down image through the mobile phone client. The PC client and the mobile client communicate with each other according to the flowchart shown in Figure 7.

## 3. Video Analysis-Based Falling-Down Detection

The falling-down detection can be accomplished with many ways [12, 13]. In this paper, we utilize the video analysis based method for falling-down detection. Actually, video analysis-based object detection has been widely used in many areas [14].

Taking into account the privacy of the elderly, the cameras in the living room and the bedroom cannot capture real-time videos. Only when the elderly falls down, the abnormal images can be viewed through the mobile client. In this paper, the falling-down detection is divided into three steps: moving human-being detection, shadow removal, and falling-down feature extraction.

### 3.1. Moving Human Being Detection

We utilize the background subtraction method [15–17] for moving human being detection. There are mainly 3 steps for background subtraction-based moving object detection: background modeling, background updating, and background subtraction. In order to get a real-time moving human being detection, we propose an improved background modeling method; combined with the Surendra background updating algorithm [18], we can get a fast accurate detection.

#### 3.1.1. Improved Background Modeling Algorithm

Background modeling refers to the extraction of the background from the video sequence, which is the key and basis step in the background subtraction algorithm. We propose an improved background modeling algorithm using the frame difference method. The core idea is to threshold the difference image to update the initial background (the initial gray value is 0) until the background is established. Considering the relatively slow motion of the elderly, the relative motion of the adjacent frames will be small or even static, which will lead to unsatisfactory results. So, in this paper, the two images doing difference operation are not adjacent frames which reflect the improvement. The core point of this algorithm is to compare the gray levels of the pixels at the same location in the two images at different times. When the threshold is less than a certain value, it is regarded as the background point. Specific steps are as follows:

Step 1. Take out five frames and every two frames have an *F* frame (*F* = 15 in this paper) separation in the original sequence. Initialize the gray value of the background image to be 0.
Step 2. Obtain the difference image from the first frame and the second frame by image subtraction. If the gray value of a pixel in the difference image is less than a distinct threshold value *T* (*T* = 20 in this paper), and at the same time the gray value of this pixel in the background image is 0, set the gray value of this pixel in the background image to the same value as the second frames *Hx*.
  (1) $$\begin{array}{c} BeiJingbuffer\left(x,y\right)=\left\{\begin{array}{cc} Hx, & \left|TempYbuffer2\left(x,y\right)-TempYbuffer1\left(x,y\right)\right|<T\&\&BeiJingbuffer\left(x,y\right)=0,\\ 0, & else. \end{array}\right. \end{array}$$
Step 3. Repeat step 2 and take the second frame and the third frame images into the calculation, until all 5 images are completed, the background image is established.
Step 4. Update the established background image in real time: make a subtraction between the current image and the background image obtained by the third step, then get the difference image. If the value of a pixel in this difference image is greater than the threshold *T*1 (here is 25), then update the value to 255. We declare this point as a moving pixel and there is no need to update the background. Otherwise, the background image needs to update to 0, which is a binary image.
  (2) $$\begin{array}{c} Rgb\_buffer=\left\{\begin{array}{cc} 255, & \left|Lumatopbuffer\left(x,y\right)-BeiJingbuffer\left(x,y\right)\right|>T1,\\ 0, & else. \end{array}\right. \end{array}$$

#### 3.1.2. The Surendra Background Update Algorithm

The background template image is the initial background image of the previous *F* ^∗^ 5 frame image, but the background image of the later frame is not static. Because of the influence of light and other objects, it is necessary to update the background template in real time to adapt to the changes of indoor light and other environmental factors. Since this step requires a background image, only the still pixels need to be updated. In this paper, we use the following weight formula to update the background, that is, the Surendra background update algorithm.

(3) $$\begin{array}{c} BeiJingbuffer\left(x,y\right)=\alpha \times Lumatobuffer\left(x,y\right)+\beta \times BeiJingbuffer\left(x,y\right). \end{array}$$

BeiJingbuffer(*x*, *y*) is one pixel of the background image BeiJingbuffer, and Lumatopbuffer(*x*, *y*) is one pixel of the current image Lumatopbuffer. *A* and *B* are the weight coefficients, which meet *α* + *β* = 1, and inequality *α* ≤ 0.5 and *β* ≥ 0.5. *α* and *β* are used to adjust the background update speed. When *α* gets larger, the background image BeiJingbuffer will adapt to the scene more quickly but will cause larger foreground noise. When *α* gets lower, BeiJingbuffer will adapt more slowly and will cause lower noise. Therefore, to a certain extent, *α* weakens the effect of foreground pixels, and *β* enhances the role of the static background pixels. In this paper, we take experience values *α* = 0.2 and *β* = 0.8.

#### 3.1.3. Neighborhood Connectivity Analysis

**Figure 8:**
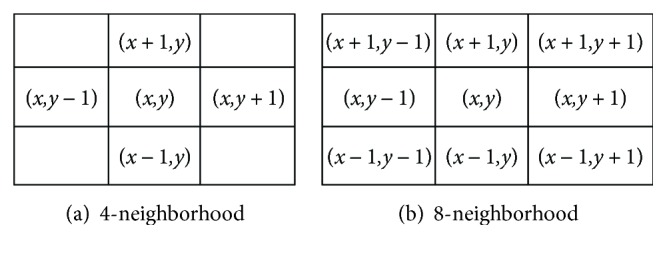
Neighborhood positions.

Connected neighborhoods are neighboring pixels that are connected to each other by some similar rule, which is often a separate area of pixels in the image. In binary maps, there are only two-pixel values of 0 and 1, so this rule is usually specified by comparing the pixel values of neighboring pixels. Commonly used neighborhood connectivity domains are 4-neighborhood and 8-neighborhood. 4-neighborhood contains the positions at the top, bottom, left, and right positions of the target pixel, which is denoted as *N*_4_ (*q*), as shown in Figure 8(a). 8-neighborhood contains the positions at the top, bottom, left, and right and the four diagonals of the target pixel, which is denoted as *N*_8_ (*q*), as shown in Figure 8(b).

In this paper, we use the 8-neighborhood for connectivity analysis, and the concrete steps are as follows:

Step 1. Scan the binary foreground image line by line and find the first foreground pixel as the target pixel, then make a mark *N*_1_.
Step 2. Label the same mark *N*_1_ for all the pixels in the 8-neighborhood area of the foreground image and do the same operation for these 8 pixels until no more foreground pixels can be found.
Step 3. Scan to the next target pixel, if this pixel has been marked, then skip to the next pixel in the same 8-neighborhood area without any operation; otherwise, make a marker *N*_2_ which is unused. Repeat step 2.
Step 4. Repeat step 3 until all the pixels in the image are scanned.

The operation of the above steps can get *M* label values *N*_1_, *N*_2_, ..., *N_M_* in the image; then, there are *M*-independent 8-connected neighborhoods. Count the size of these areas and arrange them in descending order to eliminate the area that is smaller than some distinct threshold. The background pixel values of these eliminated areas are set to 0.

**Figure 9:**
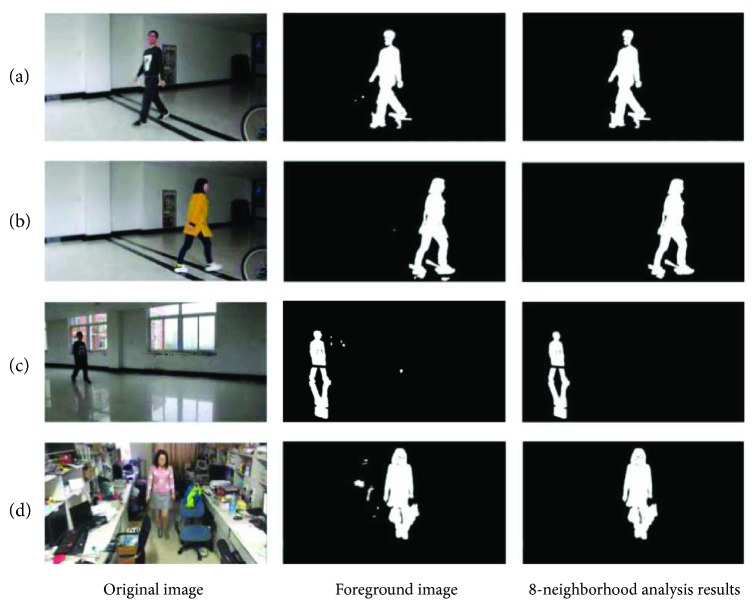
Foreground objects detection.

Several examples are shown in Figure 8 for moving human being detection. In Figure 8, there are totally four sequences. Sequences (a) and (b) have the same background under normal light conditions, but people wear clothes with different colors. Sequence (c) was captured at the laboratory corridor open space, which has white walls and light-colored tiles and the background is relatively simple, but the light is sufficient. Sequence (d) is captured near the laboratory console, which has a very complex background. The first column of Figure 9 shows the original image of the sequences, the second column shows the foreground images extracted by the Surendra background update algorithm, and the third column is the results of the foreground after the 8-neighborhood analysis. For sequences (a), (b), and (c), the moving people are detected perfectly, but shadows are also detected due to the light reflection. For sequence (d), much more noise exist in the foreground after background subtraction, the reason is that the background and the light condition are much more complex. From (a) and (b), we can notice that the dress color has little effect on the moving object detection. From the second column, we can still see that a small amount of interfering pixels exist in the segmented foreground; this is due to the environmental noise and shadowing of human motion, but they can be eliminated perfectly after an 8-neighborhood connectivity analysis, as can be seen from the third column.

### 3.2. Shadow Removal Based on HSV Color Space

After the moving object detection, the shadow caused by human occlusion and light change still exists in the binary foreground image, and it cannot be removed by the connected domain analysis. It is necessary to separate the moving human foreground pixels from the shadow pixels so as to avoid interference with the subsequent extraction of the falling-down feature. In this paper, we do the shadow removal [19–22] in the HSV color space. The HSV color space is the most commonly used color model in machine vision research. The use of the HSV color space for shadow removal is intuitive: firstly, the shadow is often caused by light change or object occlusion, so the brightness component *V* of a shadow pixel is almost lower than both of the background pixels and the foreground pixels at the same location; secondly, the color tone component *H* of a shadow pixel is almost constant; and thirdly, the saturation component S of the shadow is almost low.

For indoor applications, the cameras are fixedly installed, so the background is static. In this case, the reflection coefficient of the detected target shadow points *ρ*_t_(*x*, *y*) is equal to that of the background points *ρ*_B_(*x*, *y*), as depicted in

(4) $$\begin{array}{c} \rho_{B}\left(x,y\right)=\rho_{t}\left(x,y\right). \end{array}$$

On the other hand, the pixel brightness *S*(*x*, *y*) is a product of the light intensity *E*(*x*, *y*) and the reflection coefficient *ρ*(*x*, *y*), so we have

(5) $$\begin{array}{c} S\left(x,y\right)=E\left(x,y\right)\rho \left(x,y\right). \end{array}$$

According to (4) and (5), we can obtain the brightness ratio RE_t_(*x*, *y*) between the shadow pixel and the background pixel as follows:

(6) $$\begin{array}{c} {RE}_{t}\left(x,y\right)=\frac{S_{t}\left(x,y\right)}{S_{B}\left(x,y\right)}=\frac{E_{t}\left(x,y\right)}{E_{B}\left(x,y\right)}. \end{array}$$

According to the principle of shadow optics, the light intensity *E*(*x*, *y*) can be obtained with

(7) $$\begin{array}{c} E\left(x,y\right)=\left\{\begin{array}{ll} L_{A}+L_{P}\times \cos∠\left(n\left(x,y\right),J\right), & lighting,\\ L_{A},\;shadow, \end{array}\right. \end{array}$$

where *L*_*A*_ is the light source intensity, *J* is the light source direction, and **n** is the surface normal vector. It can be concluded from the above analysis that RE_t_(*x*, *y*) ≤ 1, which is in line with people's visual understanding of the shadow.

In the paper, we utilize the HSV shadow elimination algorithm (8) to detect the shadow points.

(8) $$\begin{array}{c} Shadow\left(x,y\right)=\left\{\begin{array}{ll} 1, & \lambda \leq \frac{F_{V}\left(x,y\right)}{B_{V}\left(x,y\right)}\leq \delta \cap \left(F_{S}\left(x,y\right)-B_{S}\left(x,y\right)\right)\leq \alpha_{S}\cap \left|F_{H}\left(x,y\right)-B_{H}\left(x,y\right)\right|\leq \alpha_{H},\\ 0, & other, \end{array}\right. \end{array}$$

where *F*_*H*_(*x*, *y*), *F*_*S*_(*x*, *y*), and *F*_*V*_(*x*, *y*) are the *H*, *S*, and *V* components of the current image *F*(*x*, *y*); *B*_*H*_(*x*, *y*), *B*_*S*_(*x*, *y*), and *B*_*V*_(*x*, *y*) are the *H*, *S*, and *V* components of the background image *B*(*x*, *y*); and Shadow(*x*, *y*) is a binary image that shows whether a pixel is a shadow point. The greater the ambient brightness is, the smaller *λ* is; *δ* is a parameter set to avoid too many points being mistaken for shadow points to enhance robustness. *λ* and *δ* satisfy 0 < *λ* < *δ* < 1. The shadows are more saturated and color change is not obvious, so 1 > *α*_*S*_ > 0. In order to test the results more satisfactory, a value *α*_*H*_ was added to the limit, which can be adjusted according to specific application scenarios.

**Figure 10:**
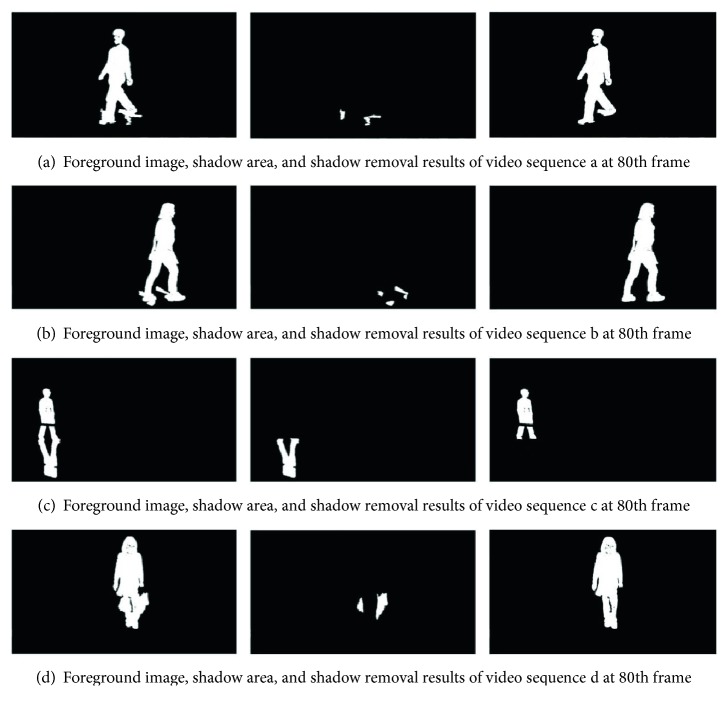
Experiment results of the shadow removal.

The effect of shadow removal is shown in Figure 10. The video sequences are the same to those in Figure 8. In Figure 9, the first column is the result after the 8-neighborhood connectivity analysis. The second column is the detected shadow, and the third column is the final foreground images after the shadow removal. We can see that the shadows are removed perfectly.

### 3.3. Falling-Down Feature Extraction

A falling-down means that the elderly falls down suddenly and does not stand up by himself in a period of time. In this paper, we use the minimum circumscribed rectangle for falling-down feature extraction.

**Figure 11:**
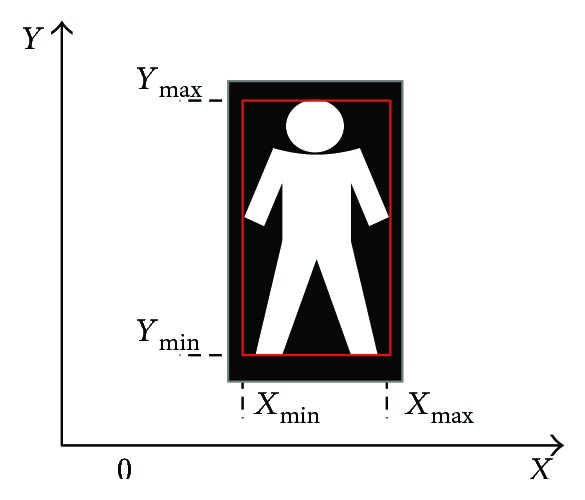
The sketch of the minimum circumscribed rectangle.

The minimum circumscribed rectangle is the rectangle including the smallest area of all the points in a certain area. For falling-down detection, this area is just the segmented foreground human body. The sketch of the circumscribed rectangle is shown in red in Figure 11.

In Figure 11, four parameters *X*_max_*, X*_min_*, Y*_max_, and *Y*_min_ are needed when plotting the rectangle, which are the minimum and maximum coordinates along the *x*-axis and the *y*-axis. As can be seen from Figure 11, *X*_min_ is the *x* position of the leftmost point in an 8-connected region, while *X*_max_ is the rightmost point in an 8-connected region. Similar meanings are with *Y*_max_ and *Y*_min_.

**Figure 12:**
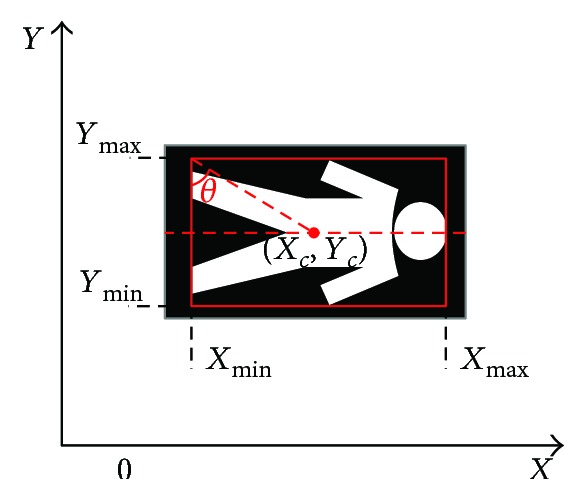
The minimum circumscribed rectangle in a falling-down state.

As shown in Figure 12, when the elderly falls down, the aspect ratio of the minimum circumscribed rectangle changes rapidly, so this aspect ratio can be used as a feature for falling-down detection. But if the elderly is too close or too far away from the camera, using the aspect ratio as the criterion will lead to a failure of detection. On the other hand, when the elderly falls, the biggest change is the center of gravity in the *x*-axis direction, while the *y*-axis direction of the center of gravity does not change much. So, in this paper, we combine the aspect ratio *K*, the absolute slope of the center of mass |*S*|, and the center of gravity in the *x*-axis direction *X*_mid_ to determine whether a falling-down occurs, which are calculated as follows:

(1) The aspect ratio *K*:
  (9) $$\begin{array}{c} K=\frac{Y_{\max}-Y_{\min}}{X_{\max}-X_{\min}}. \end{array}$$
(2) The slope of the centroid *S*:
  (10) $$\begin{array}{c} S=\frac{X_{c}-X_{\min}}{Y_{c}-Y_{\min}}, \end{array}$$

where *X_c_* and *Y_c_* are defined in

(11) $$\begin{array}{c} X_{c}=\frac{\sum_{x=X_{\min}}^{X_{\max}}x^{*}n_{x}}{n},\\ Y_{c}=\frac{\sum_{y=Y_{\min}}^{Y_{\max}}y^{*}n_{y}}{n}. \end{array}$$

In (11), *n*_*x*_ is the number of foreground pixels in the *x*th column of the object, *n*_*y*_ is the number of foreground pixels in the *y*th row, and *n* is the total number of foreground pixels.

(3) The center of gravity in the *x*-axis direction *X*_mid_:
  (12) $$\begin{array}{c} X_{mid}=\frac{\sum_{\left(x,y\right)\in H}x^{*}H\left(x,y\right)}{\sum_{\left(x,y\right)\in H}H\left(x,y\right)}. \end{array}$$

In (12), *H*(*x*, *y*) is the gray value of the moving object in the rectangle frame at pixel (*x*, *y*).

When the elderly falls, *K* < 1, |*S*| > 1, and *X*_mid_ has a big change.

**Figure 13:**
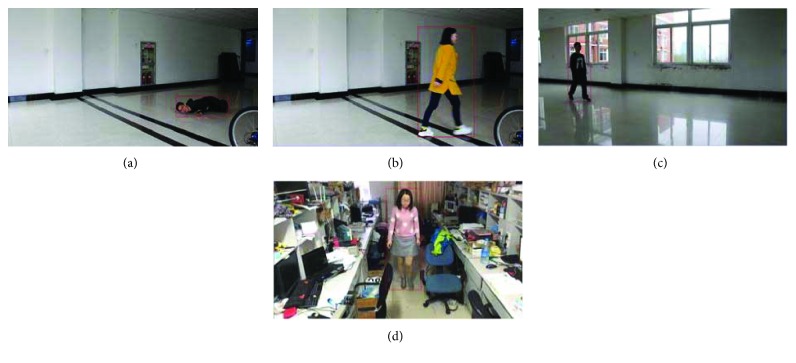
Example obtained minimum circumscribed rectangles.

Figure 13 shows several examples of the minimum circumscribed rectangles obtained according to the method proposed in this paper.

## 4. System Setup and Experimental Results

**Figure 14:**
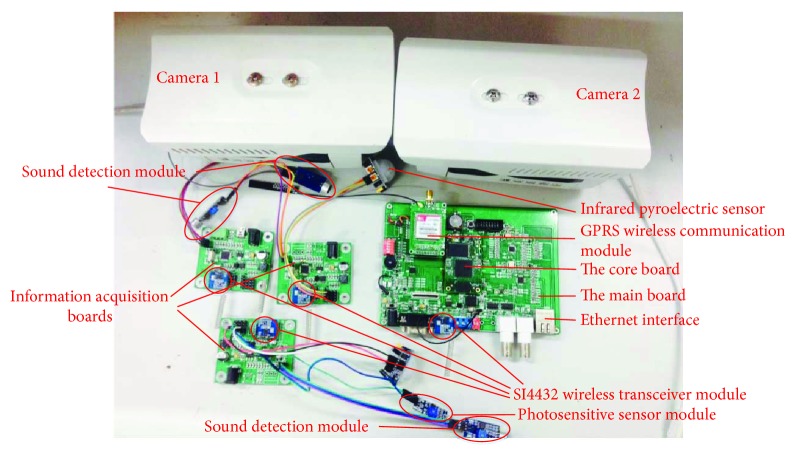
The designed system and its setup.

According to the functions of the elderly care system, we designed and implemented the whole system. As mentioned before, the system is divided into two components, the main control board and the information acquisition board. The designed embedded platform is shown in Figure 14. In Figure 14, the right-hand side is the main control board, which is used for video analysis and multi-information fusion. The left-hand side is the information acquisition boards, which are placed at the door, the toilet, and the bedroom to collect information of the elderly living information.

The hardware platform, armed with the previously described algorithms, is thoroughly tested. The experimental results are shown in Table 1.

As can be seen from Table 1, eight functions of the system were tested. In Table 1, the status “out,” “out without back,” “at home,” and “toilet abnormality” are obtained easily with the infrared pyroelectric sensor modules, through the detected moving direction of the elderly. Here, “Toilet abnormality” means that the elderly stays too long in the toilet. The “sleeping” and “sleeping abnormality” are obtained with the sound detection module mounted on the bed. A “sleeping abnormality” status means that the elderly may not breathe for a certain period of time. The “getting up” status and “falling-down” status are obtained through video analysis, as aforementioned. Experimental results show that the system meets the design requirement.

## 5. Conclusions and Discussions

In this paper, we propose an elderly care system based on multiple information fusion. Experiments demonstrate that the system can effectively notify the relatives through the GPRS when something unexpected occurs to the elderly. Moreover, the system can provide an interface for the relatives to inquire the living status of the elderly through an app installed on their mobile phone. In general, the developed system has the characteristics of quick response, high accuracy, and low cost, which can meet the requirements of real-time monitoring of the living status for the elderly and can be widely applied to the elderly care at home. In the future work, the proposed system can be further improved by integrating with other wearable sensors, for example, the ECG sensor [23], which can give much more information about the living status of the elderly.

## Figures and Tables

**Table 1:** System function test results.

Function	Total test numbers	Correct result	Accuracy rate
Out	100	100	100%
Out without back	150	150	100%
At home	100	100	100%
Toilet abnormality	150	147	98%
Sleeping	100	99	99%
Sleeping abnormality	150	146	97.33%
Getting up	100	100	100%
Falling-down	150	142	94.6%

## References

[1] Wong A. (2015). *Population Aging and the Transmission of Monetary Policy to Consumption*.

[2] Ma B. (2014). *The Monitor System of Elderly People Living Alone Based on the Comprehensive Computer Vision*.

[3] Bai Y., Li J., He J. (2014). The design of the fall detection system based on embedded video monitoring. *Television Technology*.

[4] Liu L., Stroulia E., Nikolaidis I., Miguel-Cruz A., Rios Rincon A. (2016). Smart homes and home health monitoring technologies for older adults: a systematic review. *International Journal of Medical Informatics*.

[5] Jacobsson A., Boldt M., Carlsson B. (2015). A risk analysis of a smart home automation system. *Future Generation Computer Systems*.

[6] Kidd C. D., Orr R., Abowd G. D. (1999). *The Aware Home: a Living Laboratory for Ubiquitous Computing Research International Workshop on Cooperative Buildings*.

[7] Khosla R., Chu M. T., Kachouie R., Yamada K., Yoshihiro F., Yamaguchi T. Interactive multimodal social robot for improving quality of care of elderly in Australian nursing homes.

[8] Suryadevara N. K., Mukhopadhyay S. C. (2012). Wireless sensor network based home monitoring system for wellness determination of elderly. *IEEE Sensors Journal*.

[9] Sathiyabama B., Malarkkan S. (2012). Low power adders for MAC unit using dual supply voltage in DSP processor. *International Proceedings of Computer Science & Information Tech*.

[10] Luo Z., Liu Z., Zhang J., Song C. The design of musical instrument tuning system based on stm32f103 microcomputer.

[11] Zhang Y. N., Ning H. Y., Bai J., Chen B.-C., Zhou P.-C., Zhao X.-L. Elderly safety early-warning system based on android mobile phones.

[12] Lai C. F., Huang Y. M., Park J. H., Chao H. C. (2010). Adaptive body posture analysis for elderly-falling detection with multisensors. *IEEE Intelligent Systems*.

[13] Kim S. H., Kim D. W. (2012). A study on real-time fall detection systems using acceleration sensor and tilt sensor. *Sensor Letters*.

[14] Buch N., Velastin S. A., Orwell J. A. (2011). Review of computer vision techniques for the analysis of urban traffic. *IEEE Transactions on Intelligent Transportation Systems*.

[15] Bahadir K., Serdar K. (2012). Moving object detection and tracking by using annealed background subtraction method in videos: performance optimization. *Expert Systems with Applications*.

[16] Cheng M., Gao J. (2012). *An Improved Background Modeling Method for Target Detection*.

[17] Gorur P., Amrutur B. Speeded up Gaussian mixture model algorithm for background subtraction.

[18] Gupte S., Masoud O., Martin R. F. K., Papanikolopoulos N. P. (2002). Detection and classification of vehicles. *IEEE Transactions on Intelligent Transportation Systems*.

[19] Yoneyama A., Yeh C. H., Kuo C. C. J. Moving cast shadow elimination for robust vehicle extraction based on 2D joint vehicle/shadow models.

[20] Finlayson G. D., Hordley S. D., Lu C., Drew M. S. (2006). On the removal of shadows from images. *IEEE Transactions on Pattern Analysis and Machine Intelligence*.

[21] Leone A., Distante C., Ancona N., Stella E., Siciliano P. Texture analysis for shadow removing in video surveillance systems.

[22] Rahmat R. W., Al-Tairi Z. H., Saripan M. I., Sulaiman P. S. Removing shadow for hand segmentation based on background subtraction.

[23] Yoo J., Yan L., Lee S. (2009). A wearable ECG acquisition system with compact planar-fashionable circuit board-based shirt. *IEEE Transactions on Information Technology in Biomedicine*.

